# Targeted Therapy-Resistant Melanoma Cells Acquire Transcriptomic Similarities with Human Melanoblasts

**DOI:** 10.3390/cancers10110451

**Published:** 2018-11-16

**Authors:** Lionel Larribère, Silke Kuphal, Christos Sachpekidis, Sachindra, Laura Hüser, Anja Bosserhoff, Jochen Utikal

**Affiliations:** 1 Skin Cancer Unit, German Cancer Research Center (DKFZ), 69120 Heidelberg, Germany; sachindra.sachindra@charite.de (S.); l.hueser@dkfz.de (L.H.); jochen.utikal@umm.de (J.U.); 2 Department of Dermatology, Venereology and Allergology, University Medical Center Mannheim, Ruprecht-Karl University of Heidelberg, 68167 Mannheim, Germany; 3 Institute of Biochemistry, Emil-Fischer-Center, Friedrich Alexander University Erlangen-Nuremberg, D-91054 Erlangen, Germany; silke.kuphal@fau.de (S.K.); anja.bosserhoff@fau.de (A.B.); 4 Clinical Cooperation Unit Nuclear Medicine, German Cancer Research Center (DKFZ), 69120 Heidelberg, Germany; christos_saxpe@yahoo.gr; 5 Department of Hepatology and Gastroenterology, Charité-University Medical Center Berlin, 10117 Berlin, Germany

**Keywords:** melanoblast, melanoma, iPSCs, differentiation, resistance

## Abstract

The mechanisms of adaptive and acquired drug resistance in tumors are not completely understood. So far, gene amplifications or mutations, leading to the reactivation of the MAPK or PI3K pathways have been described. In this study, we used two different methods to generate human melanoblasts: (1) via differentiation from induced pluripotent stem cells (iPSCs) and (2) via dedifferentiation from melanocytes. The melanoblast transcriptomes were then compared to the transcriptome of MAPK inhibitor-resistant melanoma cells. We observed that the expression of genes associated with cell cycle control, DNA damage control, metabolism, and cancer was altered in both melanoblast populations and in both adaptive and acquired resistant melanoma samples, compared to drug-sensitive samples. However, genes involved in antigen presentation and cellular movement were only regulated in the melanoblast populations and in the acquired resistant melanoma samples, compared to the drug-sensitive samples. Moreover, melanocyte-derived melanoblasts and adaptive resistant melanoma samples were characterized by different expression levels of certain transcription factors or genes involved in the CDK5 pathway. In conclusion, we show here that in vitro models of human melanoblasts are very important tools to comprehend the expression profiles of drug-resistant melanoma.

## 1. Introduction

Melanoma’s ability to switch phenotype accounts for the high resistance to current treatments. In particular, the use of BRAF and MEK inhibitors alone or in combination faces some challenges in the clinic [1,2,3]. Indeed, after a first potentially efficient phase, the patients will almost always develop resistance to therapy and relapse [4]. The adaptive form of resistance originates shortly after the beginning of the treatment and may set the cells in a favorable condition to develop long-term acquired resistance [5,6]. Proposed adaptive resistance mechanisms are the reactivation of ERK1/2, AKT activation, and the metabolic switch from glycolysis to oxidative phosphorylation. Additionally, a SOX2-mediated upregulation of CD24 was recently suggested to promote adaptive resistance in melanoma [7]. Mechanisms of acquired resistance, which develop on the long-term treatment with BRAF and MEK inhibitors, include, mainly, gene amplification of *BRAF* or *NRAS* and *MEK* and *PI3K* mutations, also leading to the reactivation of the MAPK or the PTEN–PI3K–AKT pathways [4,8]. In addition, a significant proportion of melanoma patients is intrinsically resistant to this treatment, and an MITF-low/NF-κB-highratio has been correlated with resistance of their tumors [9].

Because of similarities in signaling pathway activation during the establishment of tumor drug resistance and during cell lineage development, molecular signatures of neural crests or melanocyte lineage progenitors have been extensively investigated [10,11,12]. Recently, for example, Rambow et al. identified by single-cell RNAseq a neural crest stem cell signature in a subpopulation of resistant melanoma cells which developed from a patient-derived xenograft (PDX) model treated with a combination of BRAF and MEK inhibitors [13]. In addition, the pharmacological inhibition of Retinoid X Receptor Gamma (RXRg) could delay the onset of resistance in this neural crest-like cell population. Along the same line, the switch of melanoma cells to a dedifferentiated phenotype under cellular stress increases the sensitivity to ferroptosis, an iron-dependent form of cell death [14]. On the basis of these data, specifically targeting dedifferentiated tumor cells via either RXRg inhibition or enhanced ferroptosis could improve the current therapeutic approaches.

In this study, we generated human melanoblasts either by dedifferentiation of mature melanocytes or differentiation of pluripotent stem cells [15,16,17,18]. We describe how transcriptomic data from human melanoblasts could help to better understand drug resistance features of melanoma cells.

## 2. Results & Discussion

### 2.1. Two Different Approaches to Generate Human Melanoblasts

**Figure 1:**
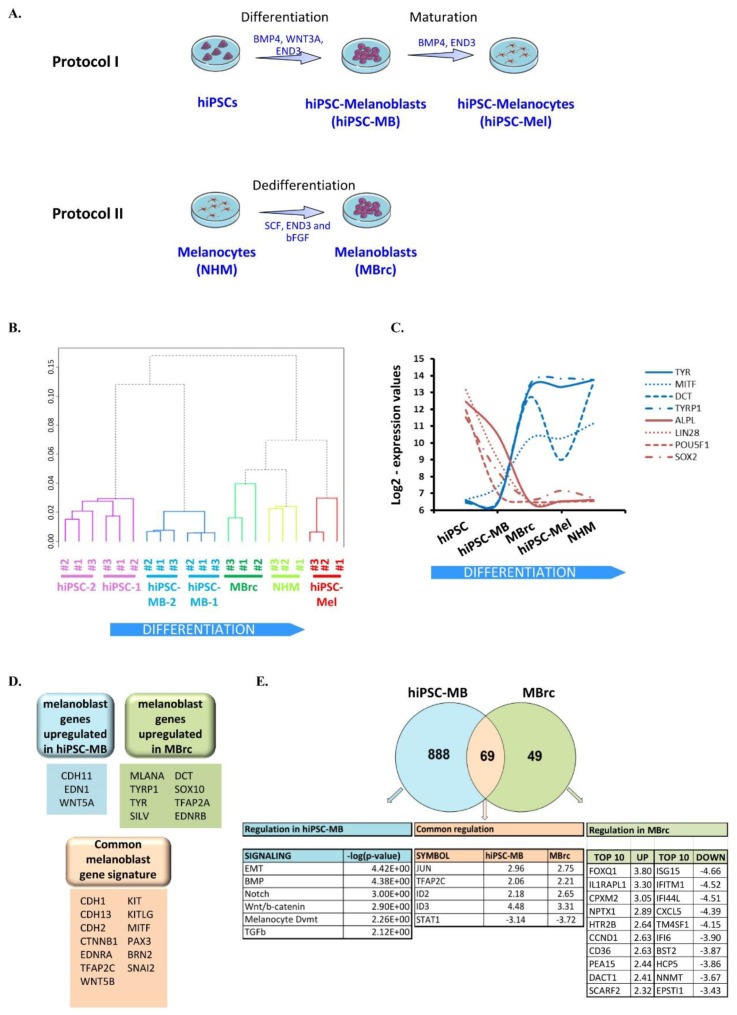
Two different approaches to generate human melanoblasts. (**A**) Schematic of two protocols to generate human melanoblasts. Protocol I leads to the directed differentiation of human induced pluripotent stem cells (hiPSCs). Protocol II leads to the dedifferentiation of human primary melanocytes (NHM). (**B**) Dendrogram of hierarchical clustering of the transcriptome of the following samples: normal human melanocytes (NHM), human induced pluripotent stem cells (hiPSC-1 and hiPSC-2), hiPSC-derived melanocytes (hiPSC-Mel), hiPSC-derived melanoblasts (hiPSC-MB-1 and hiPSC-MB-2), and melanocyte-derived melanoblasts (MBrc). The function hcluster was used, in which the parameter correlation evokes computation of Pearson-type distances. (**C**) Log2-expression values of pluripotency genes (red curves) and melanocyte-specific genes (blue curves) in the same samples as in (**B**). Biological replicates were averaged. (**D**). Identification of known melanoblast markers, which are commonly or specifically expressed in each melanoblast population. (**E**) Gene set enrichment analysis with Ingenuity (IPA) of the transcriptome of both melanoblast populations, compared to NHM samples. Log2-threshold = 2, *p*-value < 0.05. The three lists of regulated genes are provided in Table S1.

In a first step, we adapted our previous protocol of melanocyte differentiation from hiPSCs in order to identify a progenitor melanoblast stage [19]. We were able to generate human melanoblasts from two hiPSC lines (hiPSC-MB-1 and hiPSC-MB-2), by stimulation with WNT3A, EDN3, and BMP4, followed by cell sorting of KIT^+^ cells. Indeed, WNT signaling has been shown to be a key regulator of neural crest generation from hiPSCs, and EDN3 and BMP4 were described to induce KIT^+^ melanocyte precursors [15]. Moreover, KIT was suggested as a marker for melanoblasts [20]. Fully differentiated melanocytes (hiPSC-Mel) were also generated and presented a transcriptome, a morphology, and a functionality similar to those of normal human melanocytes (NHM), as previously described [19] (Figure 1A, protocol I).

The second approach was based on the dedifferentiation of mature melanocytes into progenitors (MBrc), according to the protocol of Cook et al. [18,21] (Figure 1A, protocol II). Briefly, MBrc were identified as melanocytic precursors after incubating melanocytes for 14 days in medium supplemented with SCF, EDN3, and bFGF (see Section 4 Materials and Methods). Compared to NHM, they were unpigmented, with a triangular or bipolar spindle-shaped morphology, and expressed melanoblast markers such as *SOX10*. Interestingly, *SOX10* is also described to be involved in the development of resistance to therapy, probably via a mechanism of cellular phenotype switching [22,23,24,25].

RNA samples from the melanoblast populations (hiPSC-MB-1, hiPSC-MB-2, and MBrc), from the respective parental cell lines (hiPSC-1, hiPSC-2, and NHM), and from the hiPSC-derived melanocytes (hiPSC-Mel) were analyzed for their basal gene expression profiles in an unsupervised hierarchical clustering (a dendrogram was drawn using normalized data with Pearson correlation and average linkage method). As a result, one main cluster (left) contained less differentiated cells (hiPSCs and hiPSC-MBs) and another main cluster (right) contained more differentiated cells (NHM, hiPSC-Mel, and MBrc) (Figure 1B). This result suggested that melanoblast populations generated via different methods present a slightly different transcriptome: hiPSC-derived melanoblasts cluster together with undifferentiated cells, whereas melanocyte-derived melanoblasts cluster with more differentiated cells. Accordingly, melanocyte lineage markers such as *MITF*, *TYR*, *TYRP1*, and *DCT* showed higher RNA expression in MBrc than in hiPSC-MB. Conversely, stem cell-associated genes, which were highly expressed in hiPSC, were more strongly expressed in hiPSC-MB than in MBrc (Figure 1C). These results show that hiPSC-MB samples present a less differentiated phenotype than MBrc samples. Of note, several reports associated an undifferentiated melanoma phenotype with adaptive drug resistance, and we will focus on this notion later in the text [7,26,27].

Interestingly, most described melanoblast markers were expressed (absolute expression) in both hiPSC-MB and MBrc melanoblast samples (including *MITF*, *KIT*/*KITL*, and *SNAI2*). Nevertheless, genes associated with the melanocyte lineage, such as *TYR*, *TYRP1*, *DCT*, or *SILV*, were significantly more strongly expressed in MBrc compared to hiPSC-MB. On the other hand, *CDH11*, *EDN1*, and *WNT5A* were more expressed in hiPSC-MB compared to MBrc (Figure 1D). These results confirm a common melanoblast transcriptome profile for both hiPSC-MB and MBrc samples, but with a few specific differences.

Ultimately, we analyzed the gene expression fold change of each melanoblast populations compared to NHM, and we found 69 regulated genes in common (Figure 1E and Table S1). A gene set enrichment did not show any canonical pathway; however, we observed a particular enrichment in a set of transcription factors including *JUN*, *AP2C*, *ID2*, *ID3*, and *STAT1*. As a functional validation, we have already described the crucial role of *ID3* expression in the adaptive resistance of melanoma cell lines. Indeed, we demonstrated that *ID3* silencing leads to an increased sensitivity of cells to short-term treatment with vemurafenib. In addition, we have shown that loss of *ID3* decreases melanoma cell migration and leads to the downregulation of the resistance-associated genes *MITF* and *SOX10* [28]. Additionally, *JUN* was described to play a partial role in drug-induced reprogramming and in melanoma resistance to MAPK inhibitors [29,30]. These data suggest that both melanoblast populations could be a tool of interest for the study of melanoma drug resistance.

We also found 888 regulated genes only in hiPSC-MB, which were primarily involved in signaling related to the differentiation from pluripotent stem cells (BMP, Notch, Wnt–β-catenin, TGFβ) and to the process of EMT and the development of the melanocyte lineage (*MITF*, *KIT*–*KITL*, *SOX10*, *DCT*). In addition, *FOXD1* was also upregulated in hiPSC-MB compared to NHM (Fold Change: 3.535; *p*-value < 0.05). The functional relevance of this gene during melanoma progression has been recently demonstrated by our team. Indeed, silencing of *FOXD1* in melanoma cell lines leads to a decrease in migration and invasion rates in vitro and in vivo. Moreover, *FOXD1* overexpression increases the invasion rate of melanoma cell lines, and the additional silencing of the tumor-associated gene *RAC1B* can suppress this increase. These data suggest that *FOXD1* is a strong candidate for an important role in therapy resistance of melanoma [17]. Indeed, *FOXD1* can promote drug resistance of breast cancer [31].

On the other hand, we found only 49 genes regulated in MBrc, of which the top 10 are represented in green above (Figure 1E) and in Table S1. Of note, the higher number of regulated genes in hiPSC-MB than in MBrc is consistent with their less differentiated phenotype (in comparison to NHM). In MBrc, *FOXQ1*, for example, belongs to the DNA-binding *FOX* gene family, which plays several roles during development and tumorigenesis. Interestingly, *FOXQ1* was recently described as a melanoma tumor suppressor able to mediate *MITF*-dependent melanocyte differentiation in different cellular contexts [32]. Therefore, we hypothesized that *FOXQ1* also exerts a differentiation role in melanoblasts during development.

Together, these data identify a new transcription factor gene signature in human melanoblasts. Nevertheless, the two melanoblast populations are slightly different, and hiPSC-MB show a higher number of regulated genes than MBrc when compared to NHM.

### 2.2. The Transcriptome of Melanoblasts Is Closer to Adaptive Resistant Melanoma Than to Acquired Resistant Melanoma

**Figure 2:**
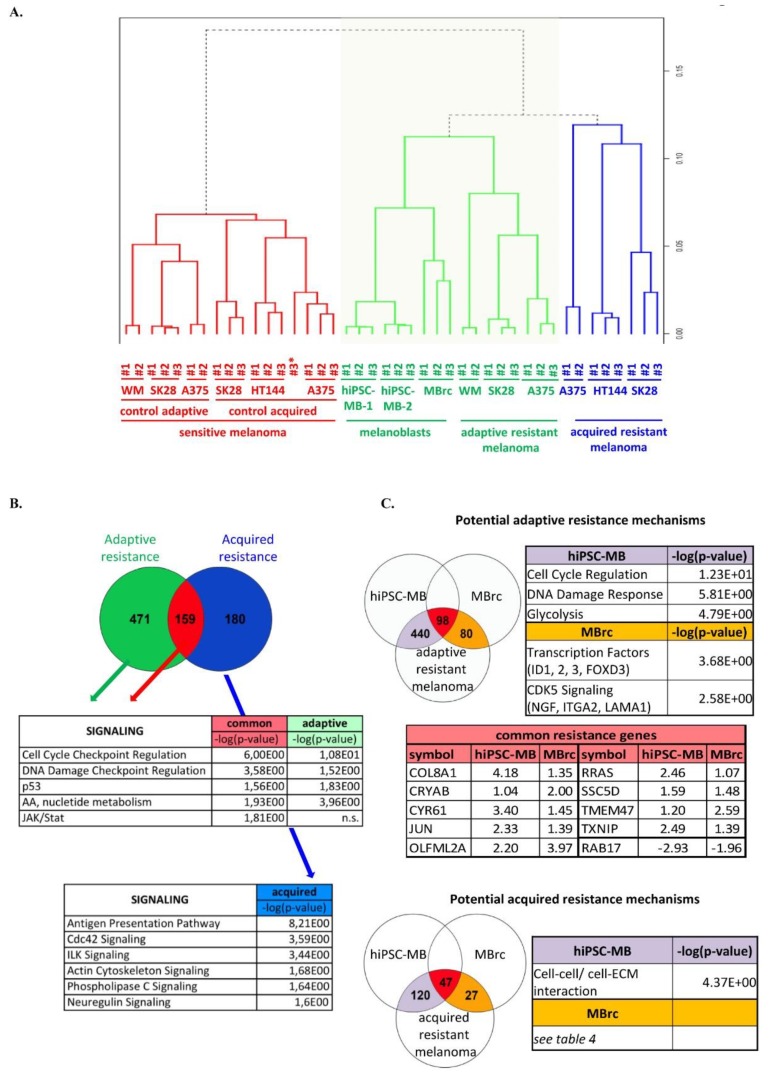
The transcriptome of melanoblasts is closer to that of adaptive resistant melanoma than to that of acquired resistant melanoma. (**A**) Dendrogram representing hierarchical clustering of the transcriptome of the two melanoblast populations (green label) and of adaptive (green label), acquired resistant (blue label), and sensitive (red label) metastatic melanoma cell lines. Note: A375#3* belongs to the control adaptive sensitive melanoma sample. The function hcluster was used, in which the parameter correlation evokes the computation of Pearson-type distances. “WM” melanoma cell line stands for “WM266-4”. (**B**) Venn diagram representing the regulated genes in either adaptive resistant melanoma samples and melanoblasts samples (green circle) or acquired resistant melanoma samples and melanoblasts (blue circle), compared to the respective sensitive melanoma samples. Gene set enrichment analysis was performed for the two gene lists with Ingenuity software (IPA). The results are presented in the tables. (**C**) Potential resistance mechanisms were investigated in both melanoblast populations in the adaptive resistance situation (top panel) or in the acquired resistance situation (bottom panel). Color code: red diagrams represent genes regulated in all groups in both adaptive and acquired situations, violet diagrams represent resistance genes regulated in hiPSC-MB only, and orange diagrams represent resistance genes regulated in MBrc only.

In a second step, we analyzed RNA samples from the two melanoblast populations, from established adaptive or acquired resistant melanoma cell lines (vemurafenib), and from the respective control samples (drug-sensitive melanoma cell lines). Of note, no significant regulation of cell death-related genes was observed in the adaptive resistant melanoma cell lines, excluding the potential cell death-related effect of vemurafenib. Strikingly, the transcriptome of all melanoblast samples clustered closer to that of drug-resistant melanoma than to that of drug-sensitive melanoma in a non-supervised hierarchical clustering (a dendrogram was drawn using normalized data with Pearson correlation and average linkage method). In addition, all melanoblast samples presented more similarities with adaptive resistant melanoma than with acquired resistant melanoma (Figure 2A). These data suggest that gene regulation program observed during the process of resistance may overlap to some extent with that observed during embryonic development. However, the overlapping ratio may vary between adaptive and acquired resistance, and this variation may be due to the acquisition of additional genetic or epigenetic alterations.

To investigate more in depth the gene regulation overlap, we performed two-group analyses, comparing all melanoblast samples and adaptive resistant melanoma samples (group 1) with sensitive melanoma control samples (group 2) (Figure 2B, green circle). In parallel, we compared all melanoblast samples and acquired resistant melanoma samples with sensitive melanoma control samples (Figure 2B, blue circle). A total of 159 genes were regulated in all melanoblast samples and all resistant melanomas (Figure 2B, red circle). A gene set enrichment analysis based on the fold change (log2-Fold Change threshold = 1) proposed pathways associated with cell cycle regulation, DNA damage regulation, cancer-associated signaling such as JAK–STAT and p53, and metabolism of amino acids and nucleotides (Table S2). Indeed, JAK–STAT and p53 signaling were already found deregulated in resistant tumor cells. Inhibitors of the p53–MDM2 axis are suggested to sensitize cells to drug-induced cell death, and a STAT3 inhibitor can overcome targeted therapy resistance [33,34]. Interestingly, potential upstream regulators of these resistance genes included MITF and CCND1. Deregulation of *MITF* has been shown to play a role in MAPK resistance in different contexts, and *CCND1* mutations or deregulation can promote drug resistance in lymphoma and myeloma [9,29,35,36,37].

A gene clustering focused on the upregulated genes in both melanoblast samples and both types of resistant melanoma samples confirmed the presence of genes involved in MAPK signaling (*MAP4K5*, *MAPKAPK2*) or in BMP signaling (*BMP1*). Several transcription factors were also identified, such as *JUND*, *FOXN3*, and *FOXO3B* (Figure S1).

On the one hand, 471 genes regulated only in melanoblasts and in adaptive resistant melanoma samples (Figure 2B, green circle) were mainly involved in mechanisms whose outcome would overlap with the aforementioned signaling pathways (Table S2). On the other hand, 180 genes regulated only in melanoblasts and in acquired resistant melanoma samples showed a tendency to control pathways involved in antigen presentation (*HLA*), cellular movement (CDC42 or ACTIN signaling), ILK, phospholipase C, or neuregulin signaling [38,39,40,41,42] (Figure 2B blue circle and Table S2).

In conclusion, these two-group analyses presented the regulation of genes related to cell cycle control, DNA damage control, cancer-associated signaling, and metabolism in both melanoblast populations and in both adaptive and acquired resistant melanoma samples. Genes regulated in both melanoblast populations and acquired resistant melanoma samples were specifically involved in antigen presentation (*HLA*), cellular movement (CDC42 or ACTIN signaling), and ILK signaling. Of note, the downregulation of *HLA-A* and *HLA-B* was described in a transcriptome analysis of acquired MAPKi-resistant tumors from melanoma patients [43].

Finally, we sought to identify the resistance-related transcriptomic differences between the two melanoblast populations. For this, we generated three gene lists based on the fold changes related to hiPSC-MB (versus parental hiPSC), MBrc (versus parental NHM), and either adaptive or acquired resistant melanoma lines (compared to the respective sensitive melanoma lines). We first focused on common mechanisms of adaptive and acquired resistance. A total of 98 genes were commonly regulated in adaptive resistance and melanoblasts, and 47 genes were commonly regulated in acquired resistance and melanoblasts (Figure 2C, red diagrams). The comparison of both gene lists is summarized in red in the table of Figure 2C. Interestingly, out of nine upregulated genes, *JUN* and *RRAS* were the only two genes also identified in the two-group analysis, and we confirmed by real-time qPCR, the upregulation of *JUN* and *RRAS* during adaptive resistance of melanoma (Figure S2). As mentioned before, *JUN* upregulation was observed in melanoma resistance to MAPKi [29]. *RRAS* (*RAS* Related), however, seems not to have been associated with therapy resistance yet, although its mutations and its deregulation have been found in many invasive tumors [44]. 

In the adaptive resistance situation, hiPSC-MB samples showed regulation of genes involved in cell cycle control, DNA damage response, and glycolysis. These signaling pathways are similar to those found in the two-group analysis and which are described as resistance mechanisms (Figure 2C, top panel) [45,46]. Changes in cellular metabolism (glycolysis) have recently been linked to tumor resistance [47]. As an example of a cell cycle-controlling gene, we confirmed the upregulation of *TFAP2A* (FC hiPSC-MB: 1.01 and FC adaptive resistant melanoma: 1.02; see Table S3) by real-time qPCR (Figure S2). MBrc samples, however, contained genes encoding for transcription factors such as *ID1*, *ID2*, *ID3*, and *FOXD3*, or involved in the CDK5 signaling pathway (*NGFR*, *ITGA2*, and *LAMA1*). We already referred to our previous work on the functional role of *ID3* in MAPK inhibitor-resistant melanoma cells and we suggest a potential role for the other family members [28]. The Forkhead Box superfamily plays a role in both neural crest development and the late stages of melanoma progression, and *FOXD3* in particular is involved in stemness maintenance of neural crest cells [48]. This suggests *FOXD3* might also regulate resistance of melanoma cells. Moreover, CDK5–NGFR signaling has been recently the focus of attention for its pro-tumorigenic function and a role in chemotherapy resistance [49,50,51]. The upregulation of all the above-mentioned genes was confirmed by real-time qPCR analysis in adaptive resistant melanoma cells (Figure S2), and the three gene lists are shown in Table S3.

In the acquired resistance situation, we observed, overall, less gene regulation (compared to the adaptive resistance situation), which suggests that melanoblast samples may be less relevant to study the long-term resistance process. hiPSC-MB samples contained regulated genes related to cell–cell and cell–ECM interaction, which are involved in cellular movement (Figure 2C bottom panel). MBrc samples contained less than a dozen regulated genes which have not yet been associated to drug resistance. These three gene lists are shown in Table S4.

## 3. Conclusions

In conclusion, we provide here a description of melanoblast-associated gene signatures, which could be involved in either adaptive or acquired resistance mechanisms in melanoma. It will be interesting in the future to complete these data with information on DNA amplification and mutation status (Next-Generation Sequencing) and on epigenetic changes, which are also factors of resistance. Furthermore, single-cell RNAseq technology could be applied to identify sub-groups of resistant tumor cells and to investigate tumor heterogeneity.

## 4. Materials and Methods

### 4.1. Cell Lines

Human melanoma cell lines (A375, HT144, SKmel 28, and WM266-4) were cultured in DMEM (Thermo Fisher Scientific, Darmstadt, Germany) with 10% FBS (Biochrom, Berlin, Germany), 0.1mM β-mercapthoethanol (Gibco, Life Technologies), 1% non-essential amino acids (NEAA), and 1% Penicilin/Streptomycin (Merck, Darmstadt, Germany). Normal human melanocytes (NHM) (PromoCell) were cultivated in melanocyte serum-free medium from PromoCell (M2 medium without PMA (Phorbol, Myristate, Acetate)). Human iPSC-1 and hiPSC-2 lines were generated from fibroblasts of healthy donors (University Medical Center Mannheim, Germany; ethics committee approval no. 2009-350N-MA) and were maintained in culture on Matrigel in mTeSR medium (Invitrogen Life Technologies, Darmstadt, Germany) as previously described [19,52]. Adaptive resistant melanoma cell lines were generated by stimulation with the BRAF inhibitor vemurafenib for 72 h at high concentration (3 μM). Acquired resistant melanoma cell lines were generated by stimulating with gradually increasing concentration of vemurafenib for six months [7,28].

### 4.2. Melanoblast Differentiation Protocols

Protocol I: Melanoblast differentiation was performed by picking hiPSC-1 and hiPSC-2 colonies and seeding them on mitomycin C-treated fibroblasts (3T3) in FAD medium composed of 2/3 DMEM, 1/3 HAM/F12 Nutrient mixture, and 10% FCS supplemented with 5 μg/mL insulin, 0.5 μg/mL hydrocortisone, 10^−10^ M cholera toxin, 1.37 ng/mL triiodothyronine, 24 μg/mL adenine, and 10 ng/mL EGF. The melanoblast specification was initiated with BMP4 (20 pM), Wnt3A (200 ng/ml), and END3 (100 nM) for 12 days. Then, KIT^+^ progenitors were FACS-sorted and directly assessed for transcriptome analysis. Alternatively, the differentiation was continued in the same medium without Wnt3A until the appearance of pigmented cells, which were selected and amplified in melanocyte culture medium (M254, Thermo Fisher Scientific, Darmstadt, Germany) supplemented with melanocyte growth factors (Invitrogen). The differentiation was performed from two independent donors.

Protocol II: MBrc were generated after incubating normal human melanocytes with MCBD 153 medium (Sigma Aldrich, St. Louis, MO, USA) containing 8% chelated FCS, 2% normal FCS (PAA Laboratories, Cölbe, Germany), 2 mM glutamine, 1.66 ng/mL cholera toxin B, 10 ng/mL SCF (Sigma Aldrich), 100 nM endothelin-3, and 2.5 ng/mL bFGF.

### 4.3. Microarray Gene Expression Profiling

Total RNA from three independent experiments was isolated with the RNeasy kit (Qiagen, Düsseldorf, Germany) from primary human melanocytes (NHM), hiPSCs, hiPSC-derived melanoblasts, NHM-derived melanoblasts, hiPSC-derived melanocytes, vemurafenib-sensitive, and vemurafenib-resistant melanoma A375, SKmel 28, WM266-4, and HT144. RNA was treated with DNase I, and RNA concentration and quality were measured by NanoDrop ND1000 spectrophotometer. cDNA was synthesized using the Revert Aid First Strand cDNA synthesis kit (Thermo Scientific, Darmstadt, Germany), according to the manufacturer’s protocol.

Labeled RNA was hybridized to whole-genome BeadChip Sentrix arrays HumanHT-12 v4 from ILLUMINA (Santa Clara, CA, USA) following the manufacturer’s indications. Microarray scanning was carried out using an iScan array scanner.

As a test for significance, a Bayes test was used on the bead expression values of the two groups of interest. The average expression value was calculated as the mean of the measured expressions of beads together with the standard deviation of the beads. After selecting the genes with *p*-values inferior to 0.05, log2-expression values of the differentially expressed genes were obtained.

## Supplementary Materials

The following are available online at http://www.mdpi.com/2072-6694/10/11/451/s1, Figure S1: Gene clustering from melanoblast samples, sensitive, adaptive and acquiredvs resistant melanoma samples; Figure S2: Real-time qPCR analysis of candidate genes’ expression: *ID1*, *ID2*, *ID3*, *FOXD3*, *NGFR*, *JUN*, and *RRAS*, in sensitive and resistant melanoma cell lines A375, SKmel28, and WM266-4. Table S1: Regulated genes in hiPSC-MB and MBrc; Table S2: Regulated genes in melanoblast and resistant melanoma; Table S3: Adaptive resistance genes in melanoblasts; Table S4: Acquired resistance genes in melanoblasts.
