# Supplementary material for: Targeted Therapy-Resistant Melanoma Cells Acquire Transcriptomic Similarities with Human Melanoblasts
Source: Cancers (Basel). 2018 Nov 16;10(11):451. doi: 10.3390/cancers10110451 (PMC6265976; doi:10.3390/cancers10110451)
Supplement: Supplementary file 1 [file cancers-10-00451-s001.zip › cancers-378747-suppl-final/cancers-378747-suppl-proofreading-done.pdf]

Supplementary materials: Targeted Therapy-Resistant Melanoma Cells Acquire Transcriptomic Similarities with Human Melanoblasts

Lionel Larribère, Silke Kuphal, Christos Sachpekidis, Sachindra, Laura Hüser, Anja Bosserhoff and Jochen Utikal

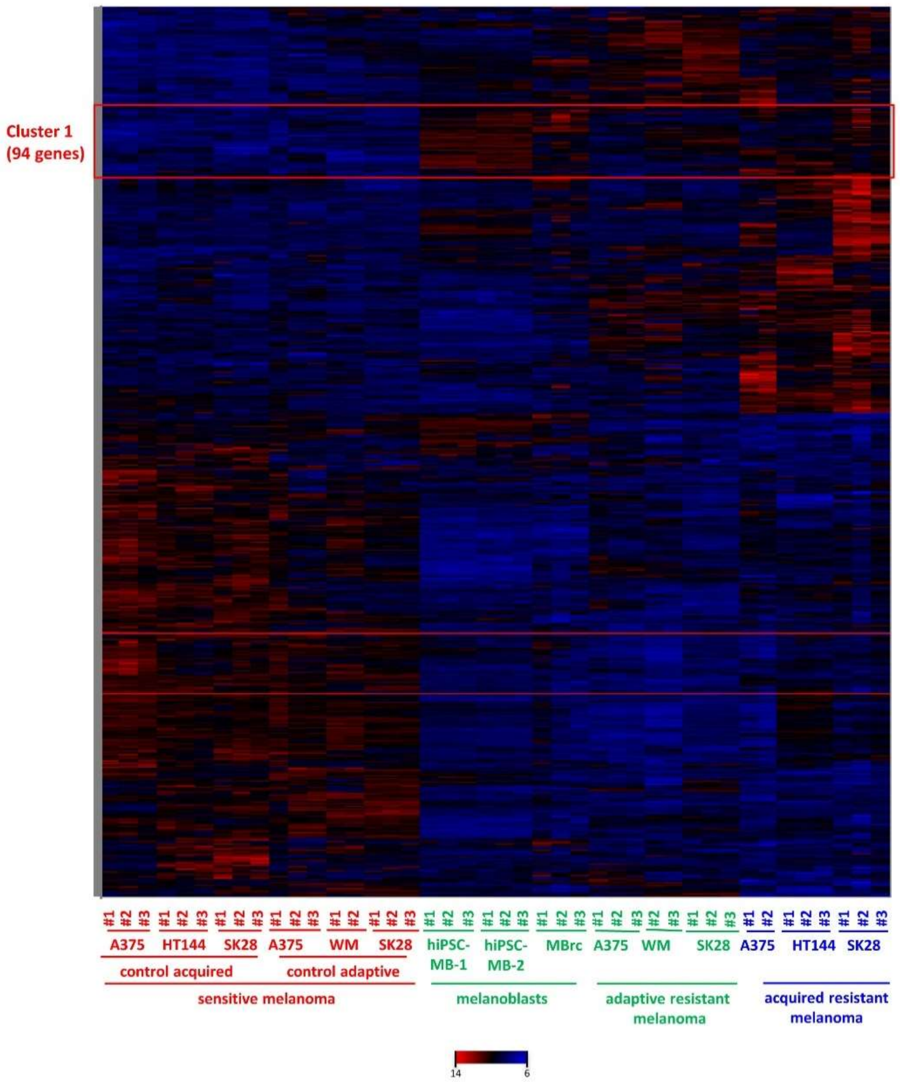

Figure S1. Gene clustering from melanoblast samples, sensitive, adaptive and acquired resistant melanoma samples. Cluster 1 represents upregulated gene in all melanoblast samples and all resistant melanoma samples.

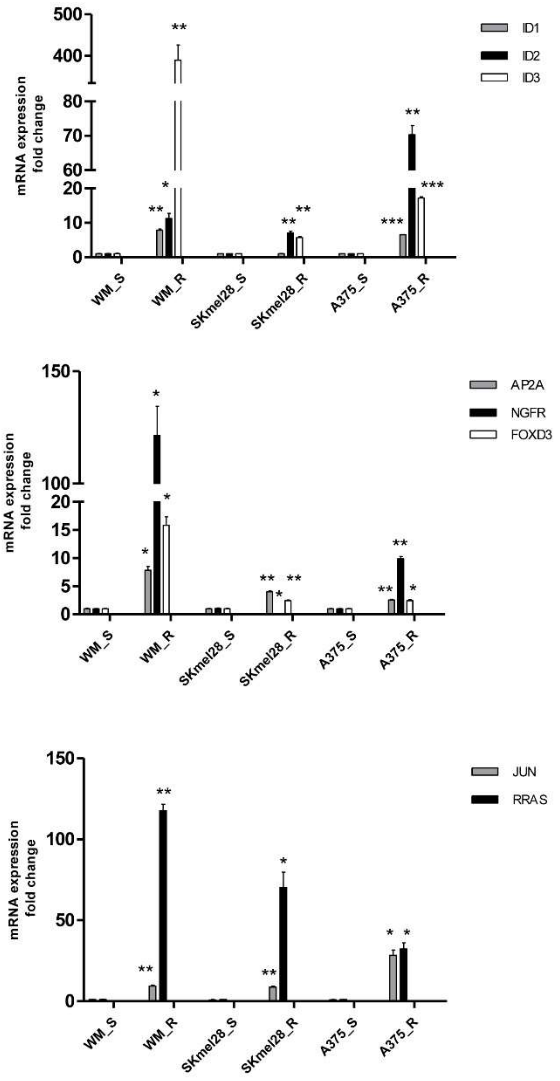

Figure S2: Real-time qPCR analysis of candidate genes' expression: *ID1*, *ID2*, *ID3*, *FOXD3*, *NGFR*, *JUN*, and *RRAS*, in sensitive and resistant melanoma cell lines A375, SKmel28, and WM266-4 Data represent a mean of three independent experiments ± SEM.
